# An Automated SeaFAST ICP-DRC-MS Method for the Determination of ^90^Sr in Spent Nuclear Fuel Leachates

**DOI:** 10.3390/molecules25061429

**Published:** 2020-03-21

**Authors:** Víctor Vicente Vilas, Sylvain Millet, Miguel Sandow, Luis Iglesias Pérez, Daniel Serrano-Purroy, Stefaan Van Winckel, Laura Aldave de las Heras

**Affiliations:** 1 European Commission, Joint Research Centre, Directorate for Nuclear Safety and Security, D-76125 Karlsruhe, Germany; Victor.VICENTE-VILAS@ec.europa.eu (V.V.V.); Sylvain.MILLET@ec.europa.eu (S.M.); Miguel.SANDOW@ec.europa.eu (M.S.); Daniel.SERRANO-PURROY@ec.europa.eu (D.S.-P.); Stefaan.VAN-WINCKEL@ec.europa.eu (S.V.W.); 2 Karlsruhe Institute for Technology, Institute for Nuclear Waste Disposal, D-76021 Karlsruhe, Germany; luis.iglesias-perez@kit.edu

**Keywords:** nuclear waste, spent nuclear fuel, ß-emitting nuclides, ^90^Sr, flow injection, ICP-DRC-MS

## Abstract

To reduce uncertainties in determining the source term and evolving condition of spent nuclear fuel is fundamental to the safety assessment. ß-emitting nuclides pose a challenging task for reliable, quantitative determination because both radiometric and mass spectrometric methodologies require prior chemical purification for the removal of interfering activity and isobars, respectively. A method for the determination of ^90^Sr at trace levels in nuclear spent fuel leachate samples without sophisticated and time-consuming procedures has been established. The analytical approach uses a commercially available automated pre-concentration device (SeaFAST) coupled to an ICP-DRC-MS. The method shows good performances with regard to reproducibility, precision, and LOD reducing the total time of analysis for each sample to 12.5 min. The comparison between the developed method and the classical radiochemical method shows a good agreement when taking into account the associated uncertainties.

## 1. Introduction

To ensure long-term safety of spent nuclear fuel, deep geologic repository is the most accepted solution by the scientific community [1]. Within this concept, the spent fuel matrix is the first barrier in case of a canister breakage [2]. Once the groundwater enters into contact with the spent fuel, the radionuclides will be released into the geosphere [2]. The Instant Release Fraction (IRF) comprises the radionuclides segregated during the irradiation and with faster dissolution rates than the matrix and constitutes one of the main sources of radiological risks for the geological repository [3]. Some of the released nuclides in this IRF are both long-lived and geochemically mobile [4].

Gamma-emitters can be easily and selectively determined using gamma-spectroscopy, but there is a lack of analytical methods for the pure ß-emitting radionuclides. The standard radiochemical methods are time-consuming and there is an urgent need for faster methods. The use of ICP-MS in nuclear decommissioning has been widely discussed in the literature [5,6] and is the preferred technique for long-lived radionuclides due to its analytical characteristics [7]. However the fact that complex matrix may create non-spectral interferences in the plasma, may limit application and makes matrix removal steps necessary [8,9,10,11]. Therefore, an efficient sample preparation protocol is needed in order to remove other elements present in the sample attaining a final solution with low total dissolved solids. The improvement of analytical procedures, involving an enhancement of selectivity and sensitivity, and shortening the time of analysis are required for the determination of radionuclides usually present at ultra-trace levels and in complex matrices.

^90^Sr is a hard to measure fission product and is of great interest due to its toxicity and high energy emission. ^90^Sr is an IRF radionuclide as it is faster released than the uranium fuel matrix [12,13]. Depending on the matrix composition of the sample, isobaric interferences by molecular or atomic ions can be expected at *m*/*z* 90 affecting the detection limit, accuracy and precision of the determination of ^90^Sr by ICP-MS. Complete removal of matrix-related interferences below background intensity is necessary to accurately quantify ^90^Sr [14]. Low instrumental detection limits (< pg·g^−1^) are required because the high natural abundance of stable Sr, present in aqueous samples, limits pre-concentration [14]. 

Pre-concentration procedures including separation are efficient solutions to overcome matrix effects and, at the same time, improve sensitivity, selectivity, and precision of the measurement. The use of flow injection methods has been proposed to overcome the tedious, time-consuming and intensive radiochemical procedures [6,15,16,17,18,19]. Flow Injection automated pre-concentration systems such as SeaFAST (ESI, Omaha, NE, USA) coupled to an ICP-MS have been successfully used [20,21] for the determination of trace metals in seawater and have demonstrated their reliability in trace metal analysis [22].

The aim of this work is to develop a rapid, selective, and sensitive method for the separation and pre-concentration of ^90^Sr in nuclear waste samples. The analytical approach uses a commercially available automated pre-concentration device (SeaFAST) coupled to an ICP-DRC-MS combining the use of a Sr-specific resin and the reaction with oxygen as reaction gas in a dynamic reaction cell (DRC) of the ICP-MS. The method has been applied for the determination of ^90^Sr at trace levels in nuclear spent fuel leachate samples without sophisticated and time-consuming procedures.

## 2. Results

### 2.1. Analytical Parameters of the SeaFAST System with ICP-DRC-MS Method

Different working ranges were tested to find the best operational conditions for the separation and preconcentration of ^90^Sr within the proposed SeaFAST system. The best results, in terms of linearity and target concentration expected in the SNF leachates, were obtained for a working range of 5 to 40 pg g^−1^.

**Figure 1:**
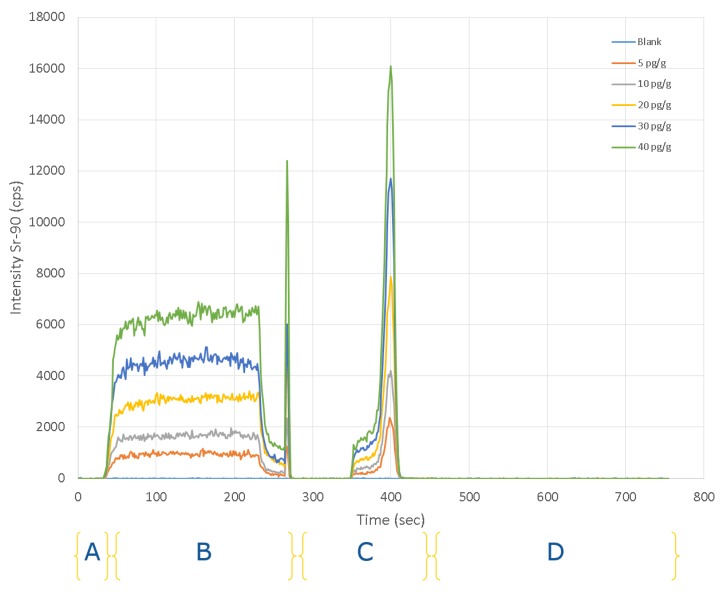
SeaFAST system with ICP-DRC-MS time resolved signal of the different ^90^Sr standards solutions. (**A**) sample load to the loops; (**B**) direct mode; (**C**) pre-concentration mode; (**D**) cleaning and pre-conditioning.

A series of standard solutions of 4 mol L^−1^ HNO_3_ containing 5 to 40 pg g^−1^ of ^90^Sr were analysed using the SeaFAST system with ICP-DRC-MS within the optimal conditions. The elution profiles obtained are reported in Figure 1. The result is a time resolved signal with 4 zones corresponding to the 4 position of the valves. The direct and pre-concentrated measurements correspond to B and C, respectively. The direct measurement (B) correlates the signal plateau height with concentration, whereas the pre-concentration measurement (C) is correlated with the signal’s area. The direct mode was used to monitor the stability of the sample signals and the correct filling of the sample loop and syringes. The ^90^Sr is eluted in a total time of 420 s and the analysis time for each sample (from injection to detection) is 12.5 min. This is significantly shorter than the conventional radiometric methods for measuring ^90^Sr, which requires at least 14 days. 

The peak of ^90^Sr in pre-concentration mode shows a fronting profile likely due to the injected volume used in the proposed method. Indeed, the peak fronting is significantly less prominent when a sample coil of 300 µL is used. However, due to the dose constraints handling radioactive samples, which require important dilution factors allowing the transfer from the hot cell to a glove-box, a 2 mL sample coil is needed. The behavior of the fronting peak seems always the same and constant within the interval of concentration used and in all samples tested having no significant influence on the final result.

**Figure 2:**
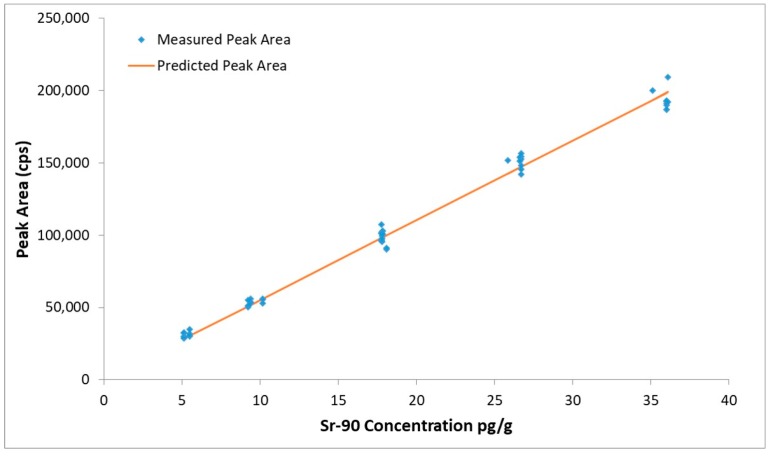
Liner regression for ^90^Sr. The interval of concentration is from 5 to 40 pg g^−1^. Blue diamonds are the data points included to calculate the linear regression. The line represents predicted values.

A calibration curve was obtained by using the peak area of ^90^Sr versus the total ^90^Sr concentration. Linear regression was calculated using the Least squares linear regression method. The calibration curve is shown in Figure 2, the method shows good linearity in the concentration interval of 5 to 40 pg g^−1^ and is suitable for the quantitative determination of ^90^Sr using the experimental conditions described in Section 4.5. 

The main analytical parameters of the proposed method are summarized in Table 1. 

The detection limit of ^90^Sr was calculated by means of repeated measurements of the blank and according to Currie [23]. The detection limit is 0.01 pg g^−1^ (that represents an absolute amount of 0.02 pg of ^90^Sr. The repeatability of the method, based on the relative standard deviation of the peak area calculated on the basis of three repetitions is always less than 1% in this concentration interval.

The intermediate precision (within-lab reproducibility) of the method, determined from results obtained on different working days with different columns and standard solutions, is around 2% for the concentration interval studied.

Nuclear spent fuel leachate analogues with a uranium concentration from 1 × 10^−7^ to 10^−5^ mol L^−1^ and spiked with different ^90^Sr concentrations were analyzed using the proposed method. Recoveries were satisfactory > 90% (±3%) in all cases. In previous experimental tests using the SeaFAST with a HR ICP-MS, the comparison of standard ^90^Sr solutions in 4 mol L^−1^ HNO_3_ and nuclear spent fuel leachate analogues with a uranium concentration from 1 × 10^−7^ to 10^−5^ mol L^−1^ spiked with ^90^Sr at different concentrations showed similar results. In fact, the calibration slopes for the different matrix compositions where compared using a heuristic approach, and no influence of the matrix could be identified. Any observed difference between various matrices can be attributed to random experimental variations (data not shown).

**Figure 3:**
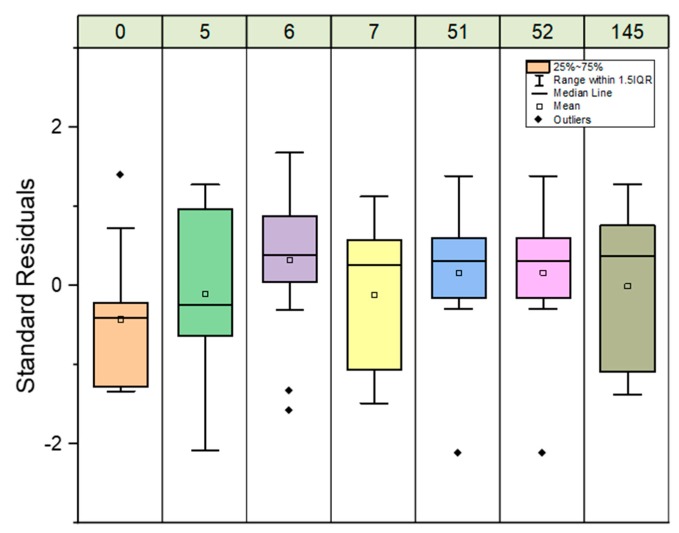
Standard residual box-whisker plots for Sr-90 reference solutions (5 to 40 pg/g) in function of time between 0 and 145 days.

The robustness of the proposed method was investigated by measuring ^90^Sr reference solutions over a long period. Figure 3 shows the standard residual box-whisker plots for the ^90^Sr references solution in function of time. The results cover ^90^Sr concentration within the linear working range from 5 to 40 pg/g. All the results are within ±2%. The method can be maintained within statistical control and is thus suitable for the determination of ^90^Sr in spent fuel leachates. 

The functional lifetime of the column depends on the number of injections that can be done with the same resin present in the column without affecting its functionality. In the present case, more than 100 analyses were performed without detecting a loss of column functionality.

### 2.2. Interferences in the Determination of ^90^Sr in the SeaFAST System with ICP-DRC-MS Detection

An exhaustive list of the main isobaric interferences in the determination of ^90^Sr by ICP-MS can be found elsewhere [15]. These interferences affect the detection limit, accuracy and precision of the determination of ^90^Sr by ICP-MS. In spent nuclear fuel leachates, the determination of ^90^Sr by ICP-MS is mainly affected by ^90^Zr. Other potential isobaric interferences such as ^58^Ni^16^O_2_^+^, ^74^Ge^16^O^+^, ^52^Cr^38^Ar^+^, ^50^V^40^Ar^+^, ^54^Fe^36^Ar^+^, ^50^Ti^40^Ar^+^, ^180^W^2+^, and ^180^Hf^2+^ can affect the determination of ^90^Sr as some transition metals are also present in the spent nuclear fuel leachates. 

**Figure 4:**
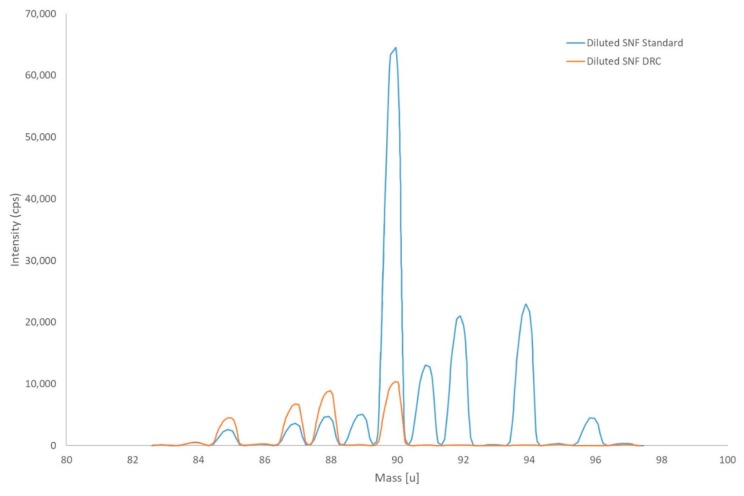
Mass spectra (from 84 till 100 u) of a diluted leachate sample; the blue spectrum shows predominantly natural Zr isotopes (90, 91, 92, 94, 96); and the presence of fission ^88^Sr as (^88^Sr/^86^Sr)meas is much bigger than (^88^Sr/^86^Sr)nat. The orange spectrum shows only the Sr isotopes and the near elimination of Zr isotopes when using the dynamic reaction cell (DRC).

A screening analysis was performed with diluted spent nuclear fuel leachate samples using the NexION 300S ICP-MS. Each sample was measured in two different dilutions. Memory effect was avoided by acid washing in between the sample measurements. The screening analyses showed different levels of natural and fission Rb, Sr, and Zr in the leachates, as demonstrated in the spectra shown in Figure 4. The presence of fission Sr (consisting of ^88^Sr + ^90^Sr only) could qualitatively be confirmed. Semi-quantitative results can be obtained but major interference corrections are needed. These corrections can be derived from the isotopic profiles of the respective elements. However, whenever these corrections become too important, the uncertainties on the calculated end results are very high. The direct and precise determination of the ^90^Sr concentration in the samples is not possible due to the high level of interference by natural zirconium coming mainly from the zircaloy. The elimination of the interference from Zr can be achieved by using the reaction of Zr with O_2_ as gas in the reaction cell of the ICP-MS (Figure 4). The reaction of Sr, Zr, and Y with O_2_ has already been reported [24] and the method applied to environmental samples [14,25]. 

**Figure 5:**
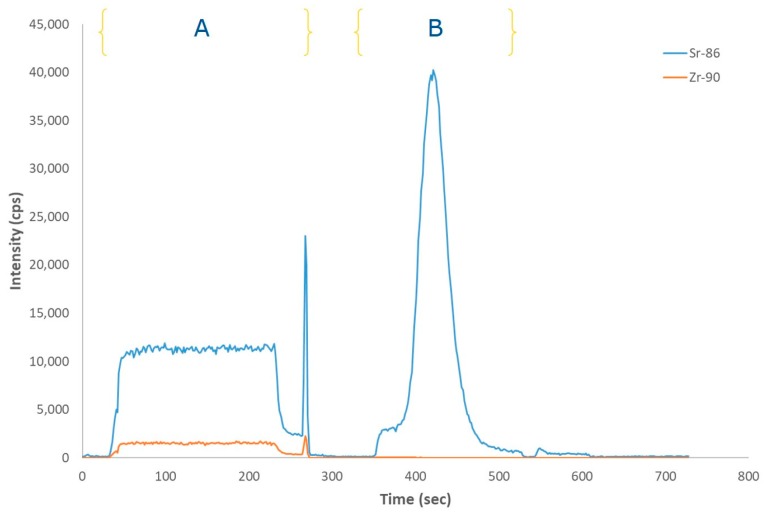
^86^Sr and ^90^Zr profiles in direct (**A**) and pre-concentration mode (**B**). The standard working solution contains 80 pg g^−1^ from an enriched standard solution of ^86^Sr and 8 µg L^−1^ of natural zirconium.

Standard working solutions of strontium and zirconium were measured using the SeaFAST system with ICP-DRC-MS detection. Figure 5 shows the elution profile of Sr in the presence of 100 times more Zr. In the direct mode (Figure 5, A) the effect of the use of O_2_ in the DRC can be observed but even if the efficiency of the reaction between Zr and O_2_ reaches 99.9%, the isobar *m*/*z* 90 is still present. In pre-concentration mode, the comparison of the elution profiles reveals the different behavior between Sr and Zr in the Sr-resin column. Sr is retained while Zr is eliminated along with the other components of the matrix (Figure 5, B). Similar results have been obtained using nuclear spent fuel leachate analogues samples. Interference free determination of ^90^Sr concentrations in the samples can be only achieved using the combination of matrix removal and DRC.

For the determination of ^90^Sr in environmental samples, low instrumental detection limits (< ng L^−1^) are required because of the high natural abundance of stable Sr. Indeed, high concentrations of stable Sr in the final sample solution require sufficient abundance sensitivity to resolve the peak tail of ^88^Sr. In spent nuclear fuel leachates, this issue is less important as the fission ^88^Sr/^90^Sr ratio is 0.8, but an efficient sample preparation protocol is needed to avoid cross-contamination with natural Sr and remove other elements present in the sample to achieve a final solution with low total dissolved solids.

### 2.3. ^90^Sr Determination in Spent Nuclear Fuel Leachates

The developed method was evaluated by analyzing different diluted spent nuclear fuel leachates and two spent nuclear fuel leachates analogues. Some samples were also analyzed by LSC. The main results of ^90^Sr analysis for three replicates are shown in Table 2. The comparison between the developed method and the classical radiochemical method shows a good agreement when considering the associated uncertainties. Indeed, the results of the analysis of spent nuclear fuel leachates analogues present a satisfactory recovery being more than 90% in all the cases. The combined uncertainty was calculated using ISO/IEC Guide 98-3:2008 version of the Guide to the Expression of Uncertainty in Measurement (GUM). The overall uncertainty was obtained by identifying, quantifying and combining all individual contributions, mainly the ^90^Sr standard reference solution (1.73%), sample and standard solutions weightings (0.054%), dilutions (0.072%), calibration (0.56%), and the sample repeatability (0.2% to 0.4%).

The results on the spent nuclear fuel leachates are in good agreement with previously reported data in spent fuel leachates [3,12,13]. The fission ^88^Sr/^90^Sr ratio remained constant at 0.8 over the whole series of measurements showing good agreement with ORIGEN code calculations (ORIGEN-ARP, 2000) [26] and previous screening analysis [3,12,13].

## 3. Discussion

A method for the determination of ^90^Sr based on the SeaFAST sample pre-concentration system with ICP-DRC-MS has been developed and tested in spent nuclear fuel leachates. The method is fully automated and provides an analysis time for each sample (from injection to detection) of 12.5 min. This is significantly shorter than the accepted radiometric method for measuring ^90^Sr. In radiometric methods ^90^Sr is normally measured through its daughter ^90^Y, requiring 2–3 weeks in-growth time to reach secular equilibrium between ^90^Sr and ^90^Y. Indeed, the on-line separation and pre-concentration of ^90^Sr from the matrix elements combined with the reaction with oxygen as reaction gas in a dynamic reaction cell (DRC) of ICP-MS removed the main interferences affecting the accurate determination of ^90^Sr in the samples. The method showed good performances with regard to the main analytical figures of merit with a LOD of 0.01 pg g^−1^ (0.04 Bq g^−1^). Although the ICP-DRC-MS is inferior to commonly used radiometric methods with respect to the minimum detectable activity (mBq level) it represents a time and cost-effective alternative technique for nuclear samples down to activities of about 1 Bq g^−1^. 

Furthermore, among other advantages the proposed method is very simple, minimizes the risk from contamination due to the limited sample handling, reduces the consumption of the chemical reagents and produces less chemical waste that it is an important aspect to consider when working with radioactive materials. The method is completely automated reducing the risk to staff when manipulating radioactive samples.

Other similar procedures have been described in the literature. An automated sequential injection separation with a flow liquid scintillation counter for on-line detection has been used for the determination of ^90^Sr in nuclear waste [27]. The Sr-resin was used for the extraction chromatographic separation and the analytical time was reduced to 40 min, while the traditional method normally required 1–3 days. A method based on HPLC with ion chromatography coupled to LSC on-line detection was also used for the determination of ^90^Sr in reactor water from the Gösgen power plant [28]. A SIA-LOV setup with an exchangeable SPE sorbent bed and an optimized procedure for sample processing for determination of ^90^Sr using ICP-MS, has been used for monitoring samples of nuclear reactor coolant [15]. The limit of detection of this procedure depended on the configuration of the employed ICP-MS and on the available volume of the sample to be analyzed. For 1 L initial sample volume, the method detection limit (MDL) value was evaluated as 2.9 fg g^−1^ (14.5 Bq L^−1^).

The source term for the mobilization of radionuclides from spent fuel typically used in the performance assessment of a geologic repository consists of two components: The instant release fraction (IRF) and the fraction released congruently with the matrix dissolution processes [29]. The IRF represents the fraction of the inventory of long-lived safety-relevant and highly mobile radionuclides, such as ^36^Cl, ^79^Se, ^99^Tc, ^126^Sn, ^129^I, and ^135^Cs that will be released upon first contact between fuel and groundwater, after breaching of all barriers. The need of reducing the uncertainties of difficult to measure radionuclides (DTM) is a continuous search for new analytical procedures, involving an enhancement of selectivity and sensitivity, and shortening the time of analysis. 

The methodology proposed in this work is under development for the determination of other important difficult to measure radionuclides such as ^135^Cs, ^126^Sn, and ^79^Se in nuclear waste characterization. Automated procedures based on the use of ICP-MS coupled to flow injection techniques for pre-concentration and/or separation of radionuclides present in spent nuclear fuel using specific stationary phases that selectively retains these key radionuclides are being tested with promising results.

**Figure 6:**
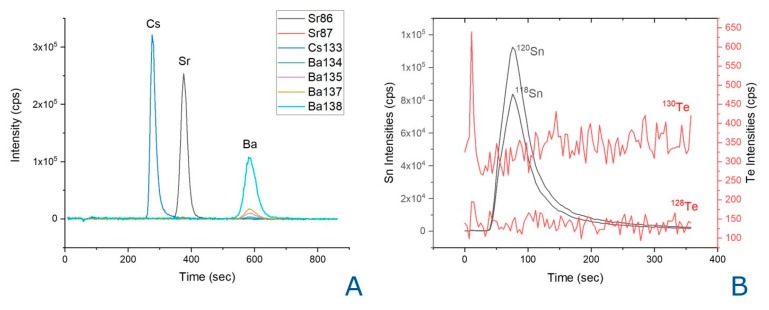
SeaFAST with ICP-DRC-MS time resolved signal of (**A**) a standard solution of Cs, Sr, and Ba (500 pg g^−1^) on the Ion Pac CS12A column using methanesulfonic acid (MSA) 20 mmol L^−1^ as eluent (**B**) in HNO3 1 *v*/*v*% using a Nobias chelate-PA1 chelating column using as eluent Acetate 0.5 mol L^−1^ pH 6.

Due to its long half-life and high fission yield, ^135^Cs (*t*_1/2_ = 2.3 Ma) is identified as a major contributor within ^79^Se to the radiological risk for geological disposal/storage facilities [30]. Measurement of ^135^Cs by mass spectrometry offers a considerable advantage because of the low specific radioactivity. However, a separation procedure is required to remove isobaric interferences from ^135^Ba for the quantification of ^135^Cs [31]. The automated SeaFAST coupled to the ICP-MS using the IonPac^®^ CS12A (Thermo Fisher Scientific Inc., Waltham, MA, USA), a selective column for the analysis of alkali and alkaline earth metals, has been tested for the determination of ^135^Cs (Figure 6A). The procedure allows the separation of Cs, Ba, and Sr in the same run showing its applicability for the determination of the three elements in spent nuclear fuel leachates and providing faster analytical results.

The major difficulty in ^126^Sn measurement in samples on spent fuel is its low concentration due to its low fission yield of 0.065%. ^126^Sn has a half-life of 230.000 years. There is a considerable lack of literature regarding the analysis of ^126^Sn, and previous studies on ^126^Sn measurements were mainly intended to confirm its half-life [32,33,34]. The content of ^126^Sn in spent nuclear fuel samples has recently been reported [35]. The determination of ^126^Sn by mass spectrometry suffers from isobaric interference of ^126^Te which cannot be resolved even with modern high resolution mass spectrometers. Therefore, if Te is not fully chemically separated, it can yield incorrect ^126^Sn concentrations in nuclear waste characterization. An analytical study carried out on TRU, TEVA, UTEVA, and DGA resins to identify a suitable solid phase extraction substrate to separate tin (Sn) from tellurium (Te) has recently been published [36]. Nobias chelate-PA1 chelating resin has been employed in an online pre-concentration system using the SeaFAST with ICP-MS detection for the analysis of Sn in spent nuclear fuel leachates. Sn and Te have a different behavior in the Nobias chelate-PA1 column (Figure 6B), Te is not retained on the column and ^126^Sn can be determined successfully, indicating the potential for the determination of Sn in spent nuclear fuel leachates. 

The accurate inventory estimation of long-lived fission products in spent nuclear fuel and high-level radioactive waste (HLW) is a major concern in the long-term safety assessment of a geological repository. Further improvement is still needed to prove the applicability of the proposed automated flow injection analysis coupled to ICP-MS for the on-line separation and/or pre-concentration on the radionuclides concerned to reduce matrix-related interferences and enhance sensitivity eliminating lengthy off-line sample preparation, providing faster analytical results and improving detection limits.

## 4. Materials and Methods 

### 4.1. Reagents and Materials

High purity PFA columns with Sr-resin^®^ 50–100 µm (Triskem International, Bruz, France) were used for the pre-concentration and analysis of ^90^Sr. 

A carrier-free ^90^Sr standard reference solution containing 2046 Bq g^−1^ ± 3.2%, k = 2 from Eckert and Ziegler was used. Stock and working standards solutions were prepared gravimetrically in 4 mol L^−1^ HNO_3_. Blanks with the same composition were also prepared.

For the preparation of all solutions, high-purity water (18.2 MΩ cm) from a Milli-Q Element system designed for ultratrace analysis (Millipore, Milford, MA, USA) was used. Nitric acid, suprapur grade from Merck (Darmstadt, Germany), was further purified using a quartz sub-boiling distillation unit. Both the water purification system and the sub-boiling distillation unit were operated in a clean room class 1000. Suprapur grade reagents from Merck (Darmstadt, Germany) as NaCl and NaHCO_3_ were used to clean materials, prepare solutions and preserve and analyse samples. Natural element standards were obtained from CPI international (Amsterdam, The Netherlands) as 1000 µg·mL^−1^ stock standard solutions. The working standard solutions were prepared gravimetrically in 1% *v*/*v* sub-boiled HNO_3_ using serial dilutions.

### 4.2. Samples

Nuclear spent fuel leachates analogues were prepared in a buffer of NaCl 19 mmol L^−1^ and NaHCO_3_ 1 mmol L^−1^ pH 8.06 with a uranium concentration from 1x10^−7^ to 10^−5^ mol L^−1^ and spiked with different concentrations of ^90^Sr. Blanks with the same composition were also prepared.

A boiling water reactor (BWR) fuel with a local burn-up of 58 GWd(tHM)^−1^ was used to perform the stability studies from irradiated fuel in contact with simplified groundwater in oxidizing conditions for geological disposal applications. The main characteristic of the UO_2_ spent nuclear fuel are summarized in Table 3.

As leaching solution a carbonated solution was used. The solution (1 mmol L^−1^ NaHCO3 and 19 mmol L^−1^ NaCl), initial pH of 8.4 ± 0.1, was initially equilibrated with air under oxidizing conditions and a normal hot cell temperature (25 ± 5) °C, as described elsewhere. 

At each sampling, unfiltered aliquots were acidified with 100 µL of concentrated HNO_3_ and diluted to prepare the diluted spent fuel leachates samples. 

### 4.3. Flow Injection Set-Up

**Figure 7:**
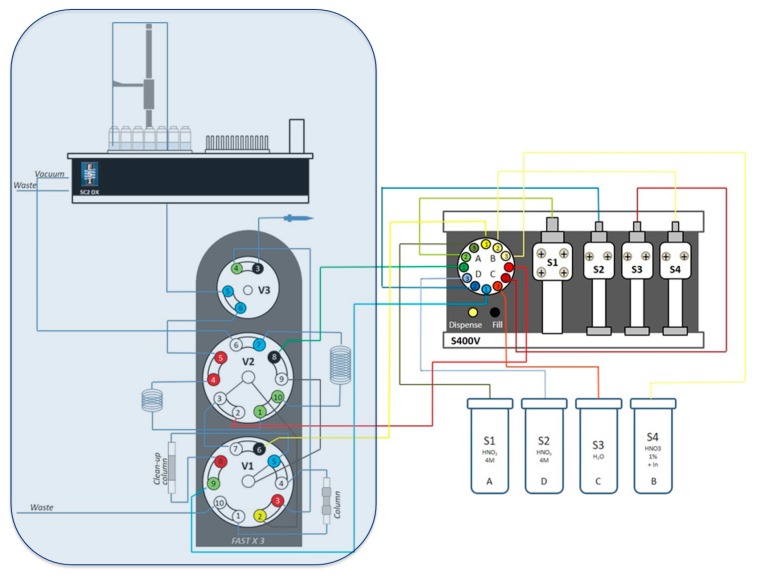
Schematic set-up of the SeaFAST system including the autosampler unit (SC2 DX), the syringe system (S400V), and valve module (FAST DX 3). Blue filled rectangle corresponds to the set-up components placed inside the glove box.

An automated system SC-2 DX SeaFAST (ESI, Omaha, NE, USA) was equipped as recommended by the manufacturer (Figure 7). The autosampler SC-2 DX was equipped with a sample probe with 1.0 mm inner diameter (ID), a vacuum pump for sample aspiration and two independent rinse pumps supplying the rinsing station with 1% HNO3 from 4 L reservoirs. The reagents were distributed by a syringe system (S400V) consisting of an ethylene chlorotrifluoroethylene (CTFE) valve with PFA rotor and four syringes: one 12 mL CTFE/polytetrafluoroethylene (PTFE) syringe (S1), and three 3 mL quartz/PFA syringes (S2, 3, and 4). The reagent flow paths were controlled by a valve module (FAST DX 3) with two 11-port CTFE valves (V1 and V2) and one 5-port CTFE valve (V3), all three with a PFA rotor. The sample coils utilized in this study had a volume of 0.6 and 2 mL. All tubing connecting the valves were made of PFA. Valve 3 was connected to the ICP-MS nebulizer. The eluent used for loading the sample was a solution of 4 mol L^−1^ HNO_3_. Samples were eluted with Milli-Q water. This system utilizes two different columns, a Nobias PA1 resin columns (200 µL) for pre-cleaning the loading eluent and a Sr-resin^®^ (200 µL) as a pre-concentration column. The system was used in both direct and pre-concentration modes.

### 4.4. ICP-MS

A nuclearized NexION 300S ICP-MS (PerkinElmer, Inc., Shelton, CT, USA) equipped with a sample introduction system consisting of a quartz cyclonic spray chamber, a type C0.5 concentric glass nebulizer and a 2 mm bore quartz injector. The ICP-MS includes a dual-channel Universal Cell and uses Dynamic Reaction Cell™ (DRC™, PerkinElmer, Waltham, MA, USA) technology. In this study ultrapure O_2_ (>99.9999%) was used as reaction gas in the collision-reaction cell (DRC). ‘Syngistix™ for ICP-MS’ software was used for defining the method set-up and data acquisition parameters. Instrument settings and conditions were optimized daily with typical values listed in Table 4. Prior to sample analysis, the NexION 300S ICP-MS was conditioned and tuned for maximum signal intensity and stability each day using a 1 µg L^−1^ standard tuning solution (PerkinElmer, Waltham, MA, USA).

Following calibration, the ICP-DRC-MS was purged with 1% prior to initial blank and sample measurements. Blanks were systematically intercalated to avoid memory effect between samples. ^90^Sr standards of 20 and 40 pg g^−1^ were analyzed after each sample sequence to verify the calibration. External calibration was used for ^90^Sr quantitation. 

### 4.5. SeaFAST Procedure

The SC-2 DX SeaFAST device was controlled using a modified protocol of the default NexION SeaFAST methods. The complete operational sequence for strontium separation, pre-concentration and elution is detailed in Table 5. Briefly, at *t* = 0, the sample is loaded onto two separate loops and injected into the system. The ICP-MS read delay (Table 5 A) is the time it takes to load the loops and for the direct mode signal to stabilize. During this time, the pre-concentration column is loaded with sample from its sample loop and the matrix ions are washed from the column to the waste. In direct mode (Table 5 B), sample is diluted online approximately 2x, mixed with In solution as internal standard to monitor correct filling of the sample loop and elution. Then it is introduced directly to the ICP-MS. After the direct mode is finished, the pre-concentrated ^90^Sr is eluted into the ICP-MS and the pre-concentration mode is acquired (Table 5 C). Following the pre-concentration elution, the column is cleaned and conditioned (Table 5 D) to prepare the column for the next sample. Although the system was optimized with a cleaning and pre-conditioning between samples, blank samples were always intercalated to avoid sample-to-sample memory effects.

### 4.6. Liquid Scintillation Counting

Liquid scintillation counting was performed using a Quantulus 1220 (PerkinElmer, Inc., Shelton, CT, USA) on ^90^Sr standard solutions, analogues and in diluted spent nuclear fuel leachates. ^90^Sr was separated on small extraction chromatography columns (Bio-rad, Hercules, CA, USA) packed with Sr-resin^®^ (50–100 µm). 0.6 mL (3 free column volumes, FCV) of the sample (in 4 mol L^−1^ HNO_3_) was gravimetrically added and the ^90^Y was fully eluted out using 1.4 mL (7 FCV) 3 mol L^−1^ HNO_3_ + 0.05 mol L^−1^ oxalic acid yielding the Y fraction. The column was further washed with 1 mL (5 FCV) 3 mol L^−1^ HNO_3_ before the Sr fraction was eluted with 2 mL 0.05 mol L^−1^ HNO_3_. A Cerenkov sample was prepared from the complete Y fraction by adding 10 mL Milli-Q H_2_O. The ^90^Sr fraction was properly mixed and then split equally into a 1 mL LSC and a 1 mL Cerenkov sample. The samples were gravimetrically added to a standard LSC counting vial and 10 mL Aqua Light scintillation liquid (LSC) or 10 mL H_2_O (Cerenkov) was added to a total filling volume of 12 mL. This sample geometry was calibrated using a ^90^Sr reference standard solution (2046 Bq g^−1^ ± 3.2%, k = 2, Eckert and Ziegler) to an efficiency of 98.8% for ^90^Sr and ^90^Y (LSC) and 66% for ^90^Y (Cerenkov). The samples were repeatedly measured with a measurement time of 20 min to roughly 92% of full ^90^Y ingrowth/decay. ^90^Sr was quantified by several methods including direct LSC and by ingrowth/decay curves of ^90^Y. The recovery of ^90^Sr was measured repeating the exact separation procedure with a spiked ^137^Cs, ^85^Sr feed. The recovery yield of ^90^Sr was determined to be 92% by direct HPGe gamma measurements of the Sr columns before and after ^90^Sr elution.

## Figures and Tables

**Table 1:** Main analytical parameters of the SeaFAST system with ICP-DRC-MS method.

Parameter	Value
Detection limit	0.02 pg
Quantification limit	0.05 pg
Regression coefficient	0.998
Repeatability (*n* = 3)	<1%
Intermediate precision	<2%
Linear working range	5 to 40 pg/g
Sensitivity	3500 cps/pg
Injection volume	2 mL
Recovery	>90%

**Table 2:** Main results on the determination of ^90^Sr in diluted spent fuel leachates using the SeaFAST system with ICP-DRC-MS detection and Liquid Scintillation Counting.

	Spent Nuclear Leachates ^1^									SNF Analogues ^2^	
Sample	54-1	54-2	54-3	54-4	54-5	54-6	54-7	54-8	54-9	SNF-18	SNF-35
**SeaFAST Method**											
Concentration pg g^−1^	41.5	78.1	64.5	16.6	16.8	20.0	30.4	24.6	17.0	18.4	36.4
u%	2.0	2.0	3.2	1.9	3.1	2.6	1.9	3.6	0.9	2.2	4.4
Recovery%										92.5	93.6
Activity ^3^ Bq g^−1^	216.0	406.8	335.8	86.3	87.4	104.0	158.5	128.0	88.6	95.7	189.8
u% ^4^	2.0	2.0	3.2	1.9	3.1	2.6	1.9	3.6	0.9	2.2	2.4
**LSC Method**											
Activity Bq g^−1^	216.0	396.5			86.0					88.6	177.7
u% ^4^	3.7	4.0			4.7					2.3	4.4
Recovery%	92	92			92					92	92

^1^ Diluted Spent nuclear leachates with dilution factors varying between 100 to 1000. ^2^ SNF analogues composition are described in Section 4.2. ^3 90^Sr specific activity is 5.21 × 10^12^ Bq g^−1^. ^4^ Combined uncertainty.

**Table 3:** UO_2_ spent nuclear fuel sample characteristics.

Segment	BWR
Total Mass (g)	2.5957
Fuel Mass (g)	2.062
Sr Inventory (µg/g)	1307
Leaching solution	Carbonated water
Contact Time (days)	0.05–315

**Table 4:** NexION^®^ 300S ICP-MS instrumental conditions.

Nebulizer	Concentric
Tripe Cone material	Pt
Spray Chamber	Cyclonic
Torch and Injector	Quartz Torch and Quartz 2.0 mm bore injector
Power (W)	1300
Plasma Gas (L·min^−1^)	16
Aux Gas (L·min^−1^)	1.2
Neb Gas (L·min^−1^)	1.03
Sample Uptake Rate (mL·min^−1^)	0.2
Isotopes	^86^Sr, ^90^Sr, ^91^Zr, ^92^Zr, ^115^In
Sweeps/Reading	5
Readings/Replicate	450
Replicates	1
Dwell Time (μs)	50
Measuring Time (s)/reading	1.6
Cell Gas	O_2_
Cell Gas Flow (mL·min^−1^)	0.5
Potential of the cell rods (RPq)	0.45

**Table 5:** SeaFAST set-up with the NexION 300S DRC ICP-MS detection.

Step	Valve Position	Syringe	Description
Load sample into the coils (A)	V1: Load V2: Load V3: Load		Sample is load into the coils. Direct measurements is 600 µL and pre-concentration in the column is 2 mL.
Direct measurement and sample load into the column (B)	V1: Load V2: Inject V3: Load	S3 180 µL·min^−1^ S4 20 µL·min^−1^ S1 2500 µL·min^−1^	Sample is eluted into the ICP-MS system. Sample is loaded on the Sr-resin^®^
Elution (C)	V1: Inject V2: Load V3: Load	S3 1000 µL·min^−1^	Pre-concentrated ^90^Sr is eluted backpressure into the ICP-MS system for online determination.
Preconditioning (D)	V1: Load V2: Load V3: Load	S3 2000 µL·min^−1^ S1 2500 µL·min^−1^	The column is cleaned and pre-conditioned
